# Patients with Dilated Cardiomyopathy (DCM) have appropriate myocardial oxygenation response to vasodilator stress

**DOI:** 10.1186/1532-429X-15-S1-O68

**Published:** 2013-01-30

**Authors:** Sairia Dass, Cameron Holloway, Joseph Suttie, Masliza Mahmod, Emily Sever, Hugh Watkins, Stefan Neubauer, Theodoros Karamitsos

**Affiliations:** 1 CMR, John Radcliffe Hospital, Oxford, UK

## Background

Microvascular dysfunction in non-ischemic DCM is well established. Despite this, little is known about whether this microvascular dysfunction is severe enough to result in ischemia on a tissue level and thus contribute to the observed derangement of cardiac energetics which is also a hallmark of DCM.

We hypothesized that in DCM the oxygen response of the myocardium to moderate stress is appropriate despite the presence of microvascular dysfunction.

## Methods

Twenty six subjects (14 DCM; 12 normal controls, table 1) were studied at 3 Tesla, (Siemens Tim Trio), with acquisition of three short-axis BOLD (using a T2-prepared sequence) and first-pass perfusion images (using a saturation recovery fast-gradient echo sequence and 0.03 mmol/kg Gd-DTPA bolus) at stress and rest (4-6 minutes i.v. adenosine, 140 μg/kg/min). Signal intensity change (SIΔ) and myocardial perfusion reserve index (MPRI) were measured from BOLD and perfusion images, respectively. LGE enhancement (SIΔ >2SD above remote myocardium) was also measured. Segments were divided according to the AHA 17 segment model.

**Table 1:** Baseline characteristics of subjects

	DCM (n=14)	Normal (n=12)	P value
Age (years)	58±9	57±9	0.92
Male, n (%)	10(71)	9(75)	0.84
Ejection fraction (%)	38±11	67±5	<0.0001
End-diastolic volume(ml)	200±74	142±42	0.02
End-systolic volume(ml)	125±65	47±14	<0.0001
LV Mass index	74±16	57±13	0.02
NYHA Class 1/2/3/4 (n)	4/6/4/0	NA	
ACE/ARB, n (%)	14(100)	0	
B-Blockers, n (%)	14(100)	0	
Spironolactone, n (%)	1(7)	0	
Digoxin, n (%)	2(14)	0	
Loop diuretic, n (%)	8(57)	0	

## Results

The baseline characteristics are summarized in table 1. During stress there were equivalent rises in rate pressure product in all groups, (normal 73±20%, DCM 74±50%, P=0.31).

In DCM, there was no significant difference in BOLD SIΔ from normals (BOLD SIΔ DCM: 16±13%, normal: 19±13%, P=0.56). For comparison, BOLD SIΔ in ischemic segments in patients with CAD was previously reported as 3.43±9.18%.

MPRI was significantly reduced in DCM (DCM: 1.52 ± 0.49; normal 1.89±0.29 P=0.03). On a segmental basis, there were no significant correlations between BOLD SIΔ and MPRI (R=0.06, P=0.43) and between BOLD SIΔ and LGE (R= 0.08, P=0.28).

## Conclusions

This is the first report of a direct comparison between myocardial perfusion and oxygenation in DCM. Our results demonstrate dissociation between perfusion and oxygenation in DCM, suggesting that the impairment of perfusion is not severe enough to cause deoxygenation during vasodilator stress.

The lack of correlation of BOLD SIΔ and LGE suggests that the development of fibrosis in DCM is not oxygen dependant.

## Funding

British heart foundation.

**Figure 1:**
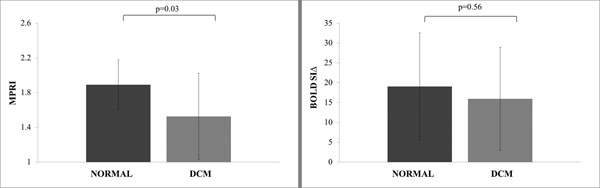
Left: MPRI mean per subject and Right: BOLD mean per subject in normal and DCM. Error bars are standard deviation.

